# miR-129-5p Promotes Osteogenic Differentiation of BMSCs and Bone Regeneration via Repressing Dkk3

**DOI:** 10.1155/2021/7435605

**Published:** 2021-07-15

**Authors:** Changming Zhao, Yulin Gu, Yan Wang, Qiaozhen Qin, Ting Wang, Meng Huang, Heyang Zhang, Yannv Qu, Jingwen Zhang, Zhangzhen Du, Xiao-Xia Jiang, Lulu Xu

**Affiliations:** ^1^Medical School of Chinese PLA, Beijing 100853, China; ^2^Department of Orthodontics, The First Medical Center, Chinese PLA General Hospital, Beijing 100853, China; ^3^Department of Endocrinology, The First Medical Center, Chinese PLA General Hospital, Beijing 100853, China; ^4^Beijing Institute of Basic Medical Sciences, Beijing 100850, China; ^5^Department of Geriatrics, Peking University Shenzhen Hospital, Shenzhen 518035, China; ^6^Anhui Medical University, Hefei 230032, China

## Abstract

**Objective:**

Accumulating evidence indicates that microRNAs (miRNAs) play crucial roles in osteogenic differentiation. However, the associated mechanisms remain elusive. This paper is aimed at exploring the role of miR-129-5p in regulating bone marrow mesenchymal stem cell (BMSC) differentiation and bone regeneration in vivo and in vitro.

**Methods:**

BMSCs were transduced by miR-129-5p mimic, miR-129-5p inhibitor, and negative control lentivirus. The ability of BMSC differentiation to osteoblast was tested by alkaline phosphatase (ALP) and alizarin red staining (ARS). The expression of osteogenic genes (Runx2, Bmp2, and OCN) was examined via quantitative RT-PCR and western blot. A mouse model of calvaria defect was investigated by Micro-CT, immunohistochemistry, and histological examination. The luciferase reporter gene assay was performed to confirm the binding between Dkk3 and miR-129-5p. For the transfection experiments, lipofectamine 3000 was used to transfect pcDNA-Dkk3 into BMSCs to overexpress Dkk3. Coimmunoprecipitation and immunofluorescent localization assay were included for exploring the role of Dkk3 and *β*-catenin.

**Results:**

miR-129-5p was induced in BMSCs and MSC cell line C3H10T1/2 cells under osteogenic medium. Overexpression of miR-129-5p significantly promoted osteogenic differentiation of BMSCs in vitro. Moreover, BMSCs transduced with miR-129-5p mimic exhibited better bone regeneration compared with BMSCs transduced with control counterpart in vivo. Luciferase and western blot data showed that Dickkopf3 (Dkk3) is a target gene of miR-129-5p and the expression of Dkk3 was inhibited in BMSCs transduced with miR-129-5p mimic but enhanced in BMSCs transduced with miR-129-5p inhibitor. In addition, Dkk3 interacted with *β*-catenin directly.

**Conclusions:**

miR-129-5p promotes osteogenic differentiation of BMSCs and bone regeneration, and miR-129-5p/Dkk3 axis may be new potential targets for the treatment of bone defect and bone loss.

## 1. Introduction

Efficient bone regeneration is widely required, since bone defects often occur due to various causes, such as tumors, infections, and bony fractures. In recent years, mesenchymal stem cells (MSCs) have been most broadly investigated and applied in transplantation and therapy both in basic experiments and in clinical trials [1]. They have been widely used in bone regeneration applications due to their self-renewal, osteogenesis capabilities, and low immunogenicity [2]. Like most stem cells, MSCs use several key transcription factors to orchestrate their proliferation and differentiation [3, 4]. In addition, more and more epigenetic regulators have been found to be involved in MSC lineage choice. Although biological functions of MSCs have been well recognized and their researches on bone tissue engineering achieved great process, there are still certain modulators undefined [5, 6].

MicroRNAs (miRNAs) are short, endogenous noncoding RNAs that function through posttranscriptional repressing specific target mRNAs to regulate various biological processes [7, 8]. The molecular mechanism of several miRNAs in the processes of bone regeneration has been investigated [9–11]. miR-7-5p induced osteogenic differentiation through repression of CRY2, which inhibited the expression of the CLOCK/BMAL1 complex [12]. miR-136-3p ameliorated alcohol-induced osteopenia with the concomitant restoration of bone mass and type-H vessel formation in a mouse model [13]. However, miR-451a accelerated bone loss via Bmp6 signaling during osteoporosis [14]. These researches indicate that the study on the functional and mechanism of osteogenesis-related miRNAs would contribute to potential strategies for bone formation. miR-129-5p has been a potential therapeutic agent because of its association with apoptosis, cell cycle, and chemoresistance [15–17]. Physical exercise is known to stimulate osteogenic commitment, and miR-129-5p expression was increased during physical exercise [18]. Though miR-129-5p was reported to promote osteoblast differentiation of human BMSCs through reducing STAT1 levels [19], the detailed mechanism of miR-129-5p in osteogenesis and bone regeneration is not completely understood.

In the present study, we have identified overexpression of miR-129-5p in BMSCs promoted their proliferation and osteoblast differentiation. Transplantation of BMSCs transduced with miR-129-5p mimic accelerated bone regeneration. Additionally, our results demonstrated that miR-129-5p exerted its effect by directly targeting the Dkk3-mediated activation of the Wnt/*β*-catenin pathway. Our study has discovered that the miR-129-5p/Dkk3 axis contributes to osteogenesis and bone regeneration, which may provide novel therapeutic strategies to maintain the stability of bone in bone defect or bone disease.

## 2. Materials and Methods

### 2.1. Animals

All experimental procedures in this study were approved and performed in accordance with the guidelines of the medical ethics committee of the Laboratory Animal Center of Beijing Institute of Basic Medical Sciences. Two- or eight-week-old male C57BL/6J mice were purchased from Beijing Vital River Laboratory Animal Technology. All mice were housed in a standard animal facility under controlled temperature (21°C) and photoperiod (12 h light/12 h dark) with free access to water and food. All in vivo experimental procedures were reviewed and approved by the medical ethics committee of the Laboratory Animal Center of the Academy of Military Medical Sciences of China.

### 2.2. Isolation and Culture of BMSCs

BMSCs were isolated from the tibia and femurs of two-week-old male C57BL/6J mice as previously described [20]. Briefly, bone marrow was flushed out with 5 ml of *α*-MEM medium (Gibco) using a syringe needle, until the bones become pale. Compact bones were carefully excised into chips and transferred in 3 ml of *α*-MEM containing 10% fetal bovine serum (FBS) in the presence of 1% collagenase II for 2 hours. The enzyme-treated bone chips were washed three times and then cultured in *α*-MEM supplemented with 10% FBS and 1% penicillin/streptomycin in a humidified incubator at 37°C and 5% CO_2_. Culture medium was replaced every 2 days. Passage was twice per week at a split ratio of 1 : 4 or 1 : 3. Cells at passages 3-4 were used for the subsequent experiments.

### 2.3. Lentivirus Transduction

Recombinant lentiviral vectors encoding for miR-129-5p mimic, miR-129-5p inhibitor, and a negative control miRNA with green fluorescent protein (GFP) were purchased from GeneChem (Shanghai, China). BMSCs were transduced as previously described [21]. Briefly, BMSCs were seeded in a six-well plate at a density of 10^5^ per well and transduced with the lentiviruses at MOI 30 (for mimic) and MOI 20 (for inhibitor and negative control) in serum-free *α*-MEM (Gibco) with HitransG A (GeneChem, Shanghai, China) for 4 hours and supplemented with medium containing 20% FBS. After 24 hours of culture, cells were replaced with *α*-MEM containing 10% FBS. GFP expression was examined by fluorescent microscopy. Subsequent experiments were carried out until the virus transduction efficiency via GFP expression was above 90%.

### 2.4. Cell Transfection

pcDNA3.1-Dkk3 and pcDNA3.1-vector were purchased from Sangon (Shanghai, China). For the transfection experiments, lipofectamine 3000 (Invitrogen, USA) was used to transfect Dkk3 or vector construct into BMSCs (pcDNA3.1: 50 nM). Transduction efficiency was analyzed by western blot.

### 2.5. Osteoblast Differentiation Induction

For osteogenic induction, BMSCs at passages 3–4 were seeded in six-well plates (1 × 10^5^ per well) with osteoblast-specific induction medium (Cyagen, China) containing 50 *μ*g/ml ascorbic acid, 1 mM dexamethasone, and 10 mM *β*-glycerophosphate. The culture medium was replaced every 2 days for a period of 14 days. Then, cells were harvested and analyzed at indicated days.

### 2.6. Alkaline Phosphatase Staining (ALP) and Activity Assay and Alizarin Red Staining (ARS)

After osteogenic induction for 7 and 14 days, the samples were fixed with 4% paraformaldehyde at room temperature for 30 minutes and then washed with PBS three times. An alkaline phosphatase kit (Abcam, Cambridge, MA, USA) was used to perform ALP staining according to the manufacturer's recommendations. ALP activity was detected using a commercial kit (# P0321S, Beyotime Biotechnology, China), and the bicinchoninic acid (BCA) method was performed to detect the protein concentration following the standard protocols.

For alizarin red staining, the mineralization tubercle was incubated with 40 mM ARS dye (pH = 4.2, Sigma, USA) for 30 minutes at 37°C and then rinsed with PBS. The stained cells in plates were photographed. Stained cells were dissolved with 10% (*w*/*v*) cetylpyridinium chloride, and the extracted solution was measured by the absorbance at 562 nm to quantify the staining. Results were performed at least three independent experiments.

### 2.7. Cell Viability Assay

Cells were seeded in 96-well plates at a density of 5 × 10^3^ cells per well. Cells were incubated in fresh medium containing 10 *μ*l of Cell Counting Kit-8 (CCK-8, Beyotime, China) at 1, 3, 5, and 7 days, respectively. The optical densities of CCK-8 were measured at 450 nm by a microplate reader (Thermo MultiskanFC). The experiment was performed three times.

### 2.8. Quantitative RT-PCR

Total RNA was extracted using the commercially available transcriptase kit (Toyobo, Osaka, Japan) with Trizol (Sigma-Aldrich) according to the manufacturer's instructions, and the total RNA was quantified using NanoDrop 2000 (Thermo). miR-129-5p determination was followed by the instruction of products (B532451 for cDNA synthesis, B532461 for PCR) from Sangon Biotech (Shanghai, China). cDNA was used as a template in quantitative PCR with SYBR Green (Toyobo) to determine specific gene expression. Reactions were run in triplicates. Relative mRNA or miRNA was normalized to endogenous *β*-actin or U6. The expression levels were calculated using the 2^-*ΔΔ*Ct^ method to determine the fold change in expression between the experimental and control groups. The miRNA and mRNA primer sequences used in this study are listed in Table 1.

### 2.9. Western Blot

Cells were first washed with PBS and lysed with RIPA buffer with complete protease inhibitor (Solarbio, Beijing, China). After protein quantification, samples were separated by 12% SDS-PAGE gel and transferred onto PVDF membranes (Bio-Rad, Hercules, CA, USA) according to the standard process. The membranes were blocked with nonfat milk and incubated at 4°C overnight with primary antibodies. Antibodies against Dkk3 (#187532), Runx2 (#23981), and Ki67 (#15580) were purchased from Abcam. Antibodies against *β*-catenin (#8480), c-Myc (#18583), and Gsk-3*β* (#12456) were purchased from Cell Signaling Technology. Antibodies against Bmp2 (AF5163), OCN (DF12303), and *β*-actin (AF7018) were obtained from Affinity Biosciences. After that, the membranes were incubated with secondary antibodies for 1 hour in room temperature, followed by TBST wash for 30 min. The blots were visualized using Tanon image scanning. ImageJ is used for quantitative analysis of the blots.

### 2.10. Luciferase Assays

The potential miR-129-5p binding sites in the Sclerostin 3′UTR were predicted with TargetScan (http://www.targetscan.org). The relationship of miR-129-5p and Dkk3 interactions was confirmed by luciferase reporter assay. The sequences that contained the wild-type (Dkk3-wt) or mutant (Dkk3-mut) seed region of Sclerostin were synthesized and cloned into a luciferase reporter plasmid. The host 293T cells were seeded in 48-well plates (1 × 10^4^ cells per well) with the indicated reporter construct and a renilla luciferase plasmid. Twenty-four hours after transfection, the activities of firefly and renilla luciferase were measured using a fluorescence spectrophotometer (Thermo MultiskanFC) according to the manufacturer's instructions. The relative transcriptional activity was normalized to the corresponding vehicle control value.

### 2.11. Coimmunoprecipitation

For immunoprecipitation analysis, cells were collected and disrupted in lysis buffer containing protease inhibitors. The total cell extracts were mixed with precleared protein G (#9007, Cell Signaling Technology, USA) and then incubated 1 *μ*g primary antibody against *β*-catenin or negative control rabbit IgG (#2729, Cell Signaling Technology, USA) at 4°C overnight. The beads were collected by centrifugation, washed three times using washing buffer, and then subjected to western blotting as described before.

### 2.12. Animal Surgical Procedure

C57BL/6J mice (8 weeks old) were randomly divided into 3 groups, including the miR-NC group (*n* = 12), miR-129-5p mimic group (*n* = 12), and miR-129-5p inhibitor group (*n* = 12). After iodophor disinfection in the dorsal region of the head, the skin and subcutaneous tissue were incised along the sagittal suture. The calvaria was exposed, and one defect with a 3 mm diameter was created on each side using a dental bur [22]. Primary closure of the incisions under sterile conditions was performed when different groups of matrigel (100 *μ*l) containing 2 million BMSC-transducted were gently implanted into the freshly formed defects. After being fed for 4 or 8 weeks with a normal diet, mice were euthanized by intraperitoneal injection of pentobarbital sodium (10 mg/kg body weight) for subsequent measurements.

### 2.13. Microcomputed Tomography (Micro-CT) Analysis

After four and eight weeks, calvarial bone was harvested and subjected to 48 hours of fixation in 4% paraformaldehyde before being analyzed by a Micro-CT imaging system (Quantum GX, PerkinElmer, Waltham, USA). Scanner parameters were set as follows: a voltage of 90 kV and current of 88 *μ*A, integration time of 14 minutes. Voxel size was selected to be isotropic and fixed at 4.5 *μ*m. The scan axis was adjusted to be normal to the subject frontal plane. Subsequently, the 3D structure of new bone formation at surgical sites was precisely evaluated using auxiliary histomorphometric software (AnalyzeDirect, Kansas, USA). Furthermore, measurements of the ratio of bone volume to tissue volume (BV/TV) and bone mineral density (BMD) of newly formed mineral were also analyzed to further identify any differences among the three groups.

### 2.14. Histological, Immunohistochemistry, and Immunofluorescence Analysis

After three-dimensional histomorphometric analysis, the calvaria samples were fixed in 4% paraformaldehyde, decalcified in 10% EDTA, washed in running water, dehydrated, diaphanization, and embedded in paraffin. Tissues were sectioned into 4 *μ*m slices for hematoxylin and eosin (H&E) staining and immunohistochemistry of Dkk3, Runx2, and OCN. Briefly described, the samples underwent treatment in a pressure cooker for 1 min at 125°C in EDTA buffer (pH 8.0) and were then cooked for another 2 min and 30 s at 100°C. The samples were allowed to cool to room temperature before incubation with 3% hydrogen peroxide for 15 min. Subsequently, the sections were incubated overnight at 4°C with primary antibody and then incubated sequentially with secondary antibody and diaminobenzidine for 20, 20, and 10 min, respectively. Finally, the slides were counterstained with hematoxylin (Abcam), dehydrated, and mounted.

For immunofluorescence analysis, tissues were cut into 6 *μ*m sections. Frozen sections were incubated for 14 h at 4°C with primary antibodies for Dkk3, Ki67, and *β*-catenin and incubated with CY3-conjugated secondary antibodies (ab6939, Abcam, MA, USA) at room temperature for 2 h. Slides were observed under a laser scanning confocal microscope (Leica, Wetzlar, Germany).

### 2.15. Statistical Analysis

Statistical analysis was performed using SPSS software (version 20.0.0.0) and GraphPad Prism 8.0 (GraphPad Software, San Diego, USA). Quantitative data was expressed as means ± SD. The statistical analysis of difference between groups was performed by Student's *t*-test. One-way analysis of variance (ANOVA) was used for comparison among more than three groups. *p* < 0.05 was considered significant.

## 3. Results

### 3.1. miR-129-5p Expression Was Induced during Osteogenic Differentiation

**Figure 1:**
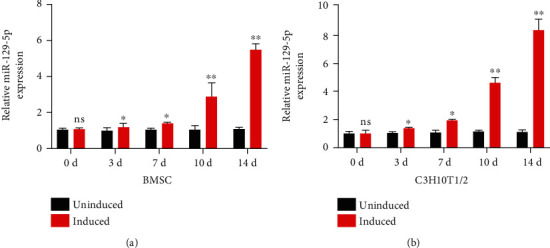
miR-129-5p expression is induced in BMSCs and C3H10T1/2 during osteogenic differentiation. BMSCs and C3H10T1/2 cells were cultured in osteogenic and normal medium. miR-129-5p expression in BMSCs (a) and C3H10T1/2 cells (b) was examined by quantitative RT-PCR at days 0, 3, 7, 10, and 14. U6 was used as an internal control. All data were expressed as means ± SD. *n* = 3 for each group in all experiments. ^∗^*p* < 0.05 and ^∗∗^*p* < 0.01.

miRNAs are critical regulators during osteogenesis and bone formation. To examine the effect of miR-129-5p on osteogenic differentiation, the expression of miR-129-5p was analyzed in BMSCs and C3H10T1/2 cells treated with osteogenic differentiation medium (Cyagen, China). As shown in Figures 1(a) and 1(b), there was a slight upregulation of miR-129-5p during the initial phase of osteoblast differentiation (day 0 to day 7), whereas the expression of miR-129-5p remarkably increased afterwards (day 7 to day 14). A time-dependent increase was found during BMSC and C3H10T1/2 cell osteogenic induction with the highest point observed at 14 days (BMSCs: 5.49 fold, *p* < 0.01; C3H10T1/2 cells: 8.35 fold, *p* < 0.01). These data indicate that miR-129-5p may be involved in osteogenic differentiation process.

### 3.2. miR-129-5p Promoted Cell Proliferation

**Figure 2:**
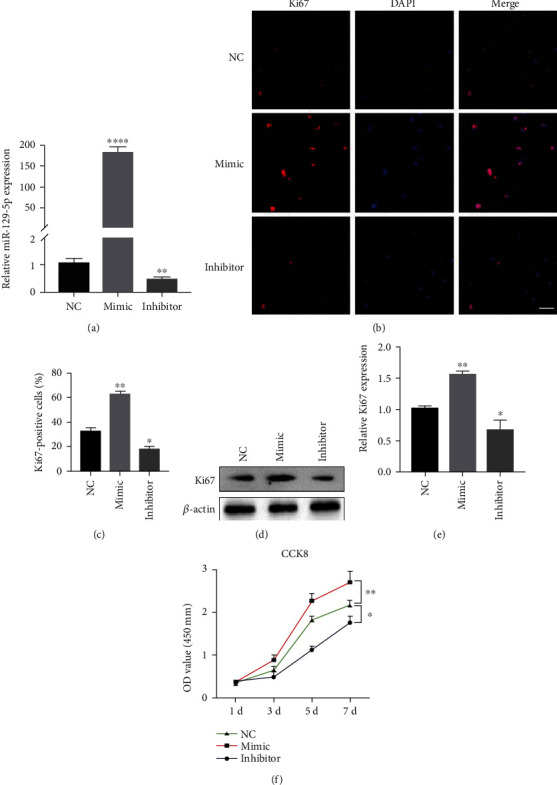
miR-129-5p promoted cell proliferation and migration. BMSCs were transduced with a lentiviral vector containing miR-129-5p mimic, miR-129-5p inhibitor, or negative control (NC) lentivirus, respectively. 48 hours after transduction, cells were used for the following analysis. (a) miR-129-5p expression was examined by quantitative RT-PCR. (b) Immunostaining with Ki67 (red) antibody and DAPI (blue) in BMSCs. Scale bars, 40 *μ*m. (c) Quantification of Ki67-positive cells in analysis was shown at (b) (*n* = 4). (d) Western blot analysis showed the expression of Ki67. (e) Quantification of Ki67 protein levels was shown at (d). (f) Cell viability was estimated with CCK-8 assay at days 1, 3, 5, and 7. (g) Images were taken at 0 h and 24 h after scratch wound. (h) Histograms represent relative wound width at 24 h normalized to that at 0 h. Values represent means ± SD of at least three independent experiments. All *p* values were based on a two-tailed *t*-test. ^∗^*p* < 0.05 and ^∗∗^*p* < 0.01.

To further investigate the involvement of miR-129-5p in osteogenesis, BMSCs were cultured and transduced with a lentiviral vector containing miR-129-5p mimic, miR-129-5p inhibitor, or control (NC) lentivirus, respectively. Transduction efficiency was confirmed by GFP expression via fluorescence microscopy analyses (data not shown) and by quantitative RT-PCR. miR-129-5p expression was significantly increased in BMSCs transduced with miR-129-5p mimic and decreased after transduced with miR-129-5p inhibitor (Figure 2(a)). Ki67 has been widely recognized as a biomarker for cellular proliferation. Immunofluorescence staining (Figures 2(b) and 2(c)) and western blot analyses (Figures 2(d) and 2(e)) indicated that the expression level of Ki67 was significantly increased in the miR-129-5p mimic group compared with that in the NC and the miR-129-5p inhibitor group. We then explored the effects of the miR-129-5p overexpression and knockdown on cell proliferation of BMSCs in vitro. Cell Counting Kit-8 (CCK-8) results indicated that proliferation abilities of BMSCs decreased by 10.70%, 23.08%, 39.30%, and 19.21% at days 1, 3, 5, and 7 posttransduction of the miR-129-5p inhibitor, as compared with the negative control miRNA (NC) (Figure 2(f), *p* < 0.05 or *p* < 0.001). Meanwhile, in BMSCs treated with miR-129-5p mimic, cell proliferation increased by 11.51%, 38.16%, 24.27%, and 24.32% at days 1, 3, 5, and 7 (Figure 2(f), *p* < 0.05 or *p* < 0.001). Collectively, these results revealed that miR-129-5p promoted cell proliferation of BMSCs.

### 3.3. miR-129-5p Enhanced Osteoblast Differentiation and Matrix Mineralization In Vitro

**Figure 3:**
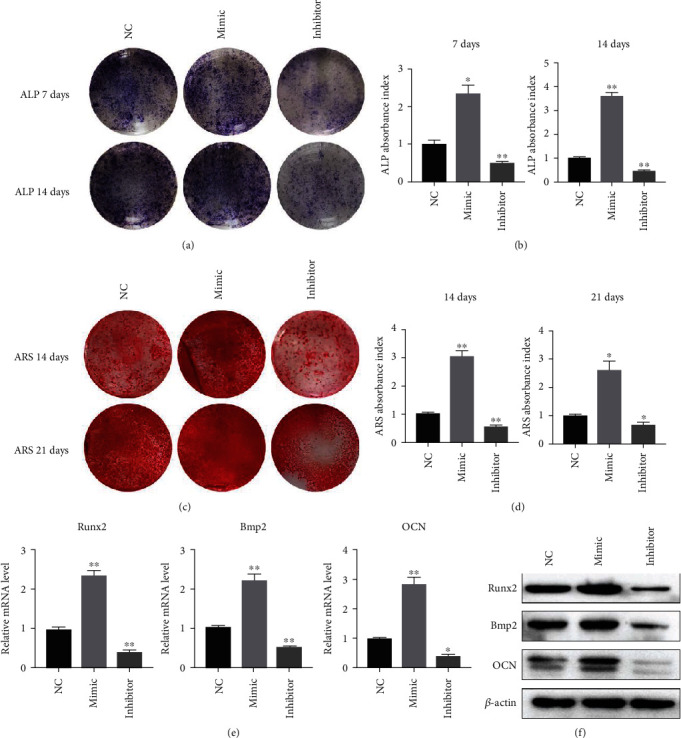
miR-129-5p promoted osteoblast differentiation of BMSCs. BMSCs were transduced with a lentiviral vector containing miR-129-5p mimic, miR-129-5p inhibitor, or negative control (NC) lentivirus, respectively. 48 hours after transduction, cells were cultured in osteoblast induction medium. ALP staining was performed at days 7 and 14 following induction (a); corresponding ALP absorbance index was calculated (b). ARS staining of BMSCs was performed at days 14 and 21 following induction (c); corresponding ARS absorbance index was calculated (d). mRNA (e) and protein (f) levels of osteoblast-related genes Runx2, Bmp2, and OCN were detected by quantitative RT-PCR and western blot, respectively. *β*-Actin was used as an internal control. All data were expressed as means ± SD. ^∗^*p* < 0.05 and ^∗∗^*p* < 0.01.

Next, we investigated the effect of miR-129-5p on the osteogenic differentiation of BMSCs. Induction was performed 72 h after lentivirus transduction. As shown in Figures 3(a) and 3(b), ALP intensity in the miR-129-5p mimic group was 2.32 fold (*p* < 0.05) and 3.60 fold (*p* < 0.01) higher than that in the NC group at 7 and 14 days, respectively. Similarly, ARS intensity in the miR-129-5p mimic group was 3.02 fold (*p* < 0.01) and 2.59 fold (*p* < 0.05) higher than that in the NC group at 14 and 21 days, respectively (Figures 3(c) and 3(d)). ALP and ARS staining data demonstrated that ALP activity and calcium deposition were significantly enhanced with miR-129-5p overexpression, whereas they were greatly attenuated with miR-129-5p knockdown. Quantitative RT-PCR and western blot assays were used to examine the expression of osteogenic genes. We found that mRNA levels of Runx2, Bmp2, and OCN were significantly higher in the miR-129-5p mimic group but lower in the miR-129-5p inhibitor group compared to their NC counterpart (Figure 3(e)). Correspondingly, protein levels of Runx2, Bmp2, and OCN were higher in the miR-129-5p mimic group but significantly lower in the miR-129-5p inhibitor group compared to their control counterpart (Figure 3(f)). Taken together, our results suggested that miR-129-5p played a positive regulatory role in osteoblast differentiation and subsequently promoted mineralization.

### 3.4. miR-129-5p Accelerated Bone Regeneration in a Mouse Model of Calvaria Defect

**Figure 4:**
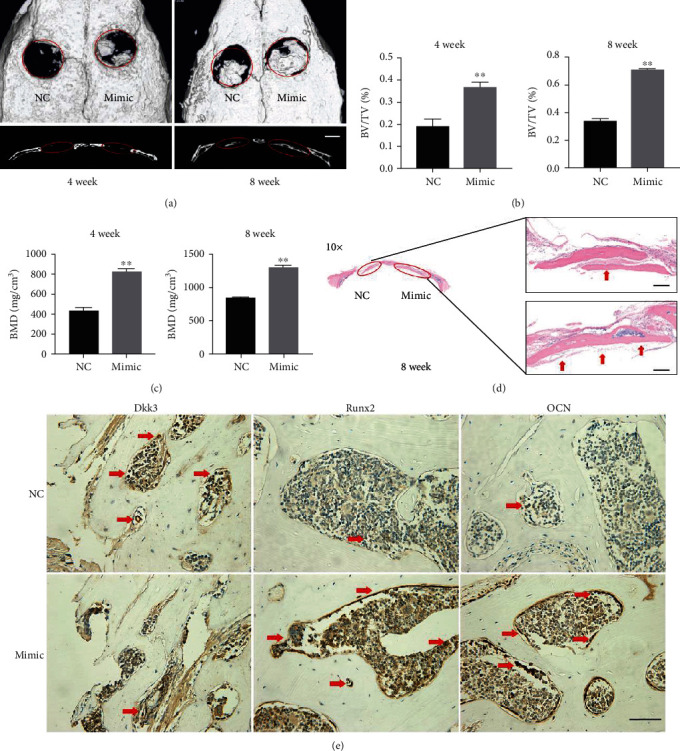
miR-129-5p enhanced bone regeneration in a mouse model of calvaria defect. BMSCs transduced with lentivirus containing miR-129-5p mimic or control miRNA (NC) were loaded on matrigel scaffolds, respectively, and then implanted into the defect of murine calvarium. Mice were sacrificed at week 4 and week 8 after implantation, and then, skull samples were collected for analyses. (a) Image with three-dimensional reconstruction (coronal and sagittal sections) from Micro-CT revealed different regeneration effects. Quantitative analysis of the 3D images, including new bone volume relative to tissue volume (BV/TV) (b) and the bone mineral density (BMD) (c), was shown. (d) Representative image of H&E staining (red arrow represented bone-like structures). (e) Immunohistochemical assessment of Dkk3, Runx2, and OCN in the NC group and the miR-129-5p mimic group (red arrow represented the positive cells). ^∗∗^*p* < 0.01.

To further explore the function of miR-129-5p on bone formation, a mouse model of calvaria defect was used. BMSCs transduced with lentivirus containing miR-129-5p mimic or control miRNA were loaded on matrigel scaffolds, respectively, and then implanted into the defect of murine calvarium. Mice were sacrificed at weeks 4 and 8 after implantation, and then, skull samples were collected for Micro-CT analyses. From coronal and sagittal views, new bone regeneration was detected in each group within the defect area (diameter: 3 mm). BMSC overexpression with miR-129-5p dramatically enhanced new bone formation compared to the control group (Figure 4(a)). Bone histomorphometrical analysis revealed that bone formation-related parameters bone volume/total bone volume (BV/TV) and bone mineral density (BMD) were increased. For accurate computation of the data, a quantitative measurement of the bone generation-related parameters was conducted. The BV/TV in the miR-129-5p mimic group (4 weeks: 0.366 ± 0.011; 8 weeks: 0.702 ± 0.027) was much higher than that in the NC group (4 weeks: 0.188 ± 0.034; 8 weeks: 0.331 ± 0.015) (Figure 4(b)). Consistently, the BMD in the miR-129-5p mimic group (4 weeks: 816 ± 26 g/cm^3^; 8 weeks: 1296 ± 53 g/cm^3^) was also significantly higher than the control counterpart (4 weeks: 428 ± 17 g/cm^3^; 8 weeks: 836 ± 22 g/cm^3^) (Figure 4(c)). Histomorphometrical analysis has been conducted by H&E staining at 8 weeks. As shown in Figure 4(d), more bone-like structures and collagen deposits were observed in BMSCs with the miR-129-5p overexpression group. In addition, immunohistochemistry data showed that osteogenic genes Runx2 and OCN were highly expressed in the miR-129-5p mimic transduction group (Figure 4(e)). Thus, miR-129-5p upregulation has a positive effect on bone regeneration.

### 3.5. Knockdown of miR-129-5p Suppressed Bone Regeneration

**Figure 5:**
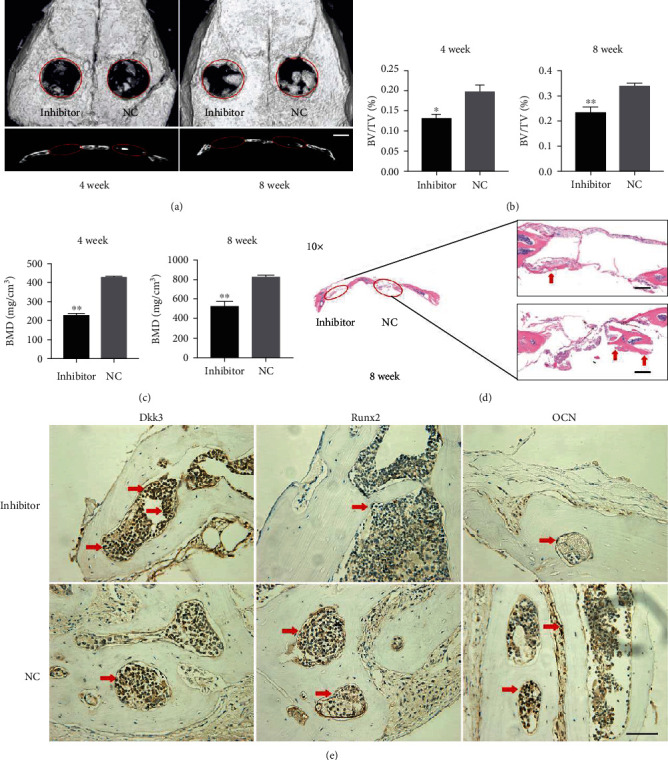
Knockdown of miR-129-5p suppressed bone regeneration. BMSCs transduced with lentivirus containing miR-129-5p inhibitor or control miRNA (NC) were loaded on matrigel scaffolds, respectively, and then implanted into the defect of murine calvarium. Mice were sacrificed at week 4 and week 8 after implantation, and then, skull samples were collected for analyses. (a) Image with three-dimensional reconstruction (coronal and sagittal sections) from Micro-CT revealed different regeneration effects in the miR-129-5p inhibitor group and the NC group. Quantitative analysis of the 3D images, including new bone volume relative to tissue volume (BV/TV) (b) and the bone mineral density (BMD) (c), was displayed. (d) Representative image of H&E staining (red arrow represented bone-like structures). (e) Immunohistochemical assessment of Dkk3, Runx2, and OCN in the miR-129-5p inhibitor group and the NC group (red arrow represented the positive cells). ^∗^*p* < 0.05 and ^∗∗^*p* < 0.01.

To further confirm the above observations in vivo, BMSCs transduced with lentivirus containing miR-129-5p inhibitor or control miRNA (NC) were transplanted into the defect of murine calvarium. As shown in Figure 5(a), miR-129-5p inhibitor repressed new bone formation compared with the NC group. 3D reconstruction revealed that new bone formation in the miR-129-5p inhibitor group was worse than that in the NC group from both the coronal and sagittal views. BV/TV decreased in the miR-129-5p inhibitor group (4 weeks: 0.131 ± 0.007; 8 weeks: 0.233 ± 0.016) compared with that in the NC group (4 weeks: 0.195 ± 0.023; 8 weeks: 0.338 ± 0.035) (Figure 5(b)). BMD in the miR-129-5p inhibitor group (4 weeks: 234 ± 33 g/cm^3^; 8 weeks: 534 ± 42 g/cm^3^) also showed the same relative effects as BV/TV compared to that in the NC group (4 weeks: 426 ± 19 g/cm^3^; 8 week: 823 ± 27 g/cm^3^) (Figure 5(c)). H&E staining further confirmed that less newly formed bone tissues were present along the defect margin in the miR-129-5p inhibitor group than that in the NC group (Figure 5(d)). Furthermore, immunohistochemistry data showed that osteogenic genes Runx2 and OCN were significantly lower in the miR-129-5p inhibitor transduction group (Figure 5(e)). Taken together, these findings further confirmed the positive effect of miR-129-5p on in vivo bone regeneration.

### 3.6. Dkk3 Is a Direct Target of miR-129-5p

**Figure 6:**
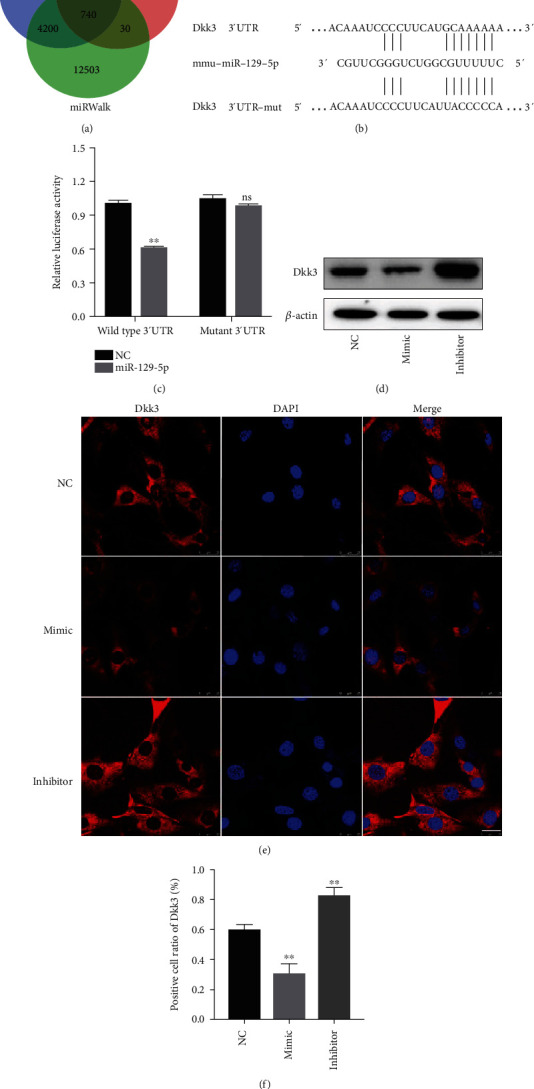
Dkk3 is a direct target of miR-129-5p. (a) A diagram showed potential targets of miR-129-5p analyzed by TargetScan, miRDB, and miRWalk. (b) Bioinformatics prediction of miR-129-5p binding sites in the 3′UTR of Dkk3 mRNA. (c) Luciferase activities of reporters containing the 3′UTR of Dkk3 in miR-129-5p-transduced, miR-129-5p-mutant-transduced, or negative control 293T cells. The relative luciferase activity in 293T cells cotransfected with negative control was designated as 1. (d) Protein levels of Dkk3 were examined by western blot. (e) Representative image of immunofluorescence staining with Dkk3 (red) antibody and DAPI (blue) in BMSCs transduced with the indicated lentivirus. Scale bars, 25 *μ*m. (f) Quantification of Dkk3-positive cells in analysis was shown at (f) (*n* = 4). ^∗^*p* < 0.05 and ^∗∗^*p* < 0.01.

To decipher the downstream genes regulated by miR-129-5p, we predicted possible targets of miR-129-5p with the help of TargetScan, miRDB, and miRWalk (Figure 6(a)). As a result of the analyses, we selected Dkk3, a related regulator of the Wnt signaling pathway which should have close connection with osteogenesis.  Figure 6(b) shows miR-129-5p binding sites in the 3′UTR of Dkk3 mRNA. Moreover, we conducted luciferase reporter assay to confirm the interaction of miR-129-5p and Dkk3. We transfected constructs into 293T cells, and a marked abatement of luciferase activity was observed in cells cotransfected with wild-type Dkk3 3′UTR and miR-129-5p at 36 h (0.60 fold, *p* < 0.01) but not in cells transfected with mutant 3′UTR. Results showed that the luciferase activity of Dkk3 3′UTR was significantly decreased in miR-129-5p-transfected 293T cells (Figure 6(c)). These findings suggested that the putative miR-129-5p binding sites located in the 3′UTR of Dkk3 mRNA are functional miR-129-5p regulatory sites. For the purpose of additional clarification of the connection existing between miR-129-5p and Dkk3, preliminary experiments using western blot showed that the protein level of Dkk3 was changed. As shown in Figure 6(d), western blot results revealed that the Dkk3 level significantly decreased by miR-129-5p mimic and increased by miR-129-5p inhibitor (*p* < 0.05 or *p* < 0.01). Immunofluorescence staining assay also showed that the miR-129-5p mimic group had the lowest ratio of cells positive for Dkk3 staining, whereas the area of Dkk3 staining positive cells was the largest in the miR-129-5p inhibitor group (Figures 6(e) and 6(f)). Consistently, the expression of Dkk3 in new bone was lower in the miR-129-5p mimic group but higher in the miR-129-5p inhibitor group (Figures 4(e) and 5(e)). Collectively, these findings confirmed that Dkk3 was a direct target of miR-129-5p.

### 3.7. miR-129-5p Regulated BMSC Osteogenic Differentiation via Repressing Dkk3

**Figure 7:**
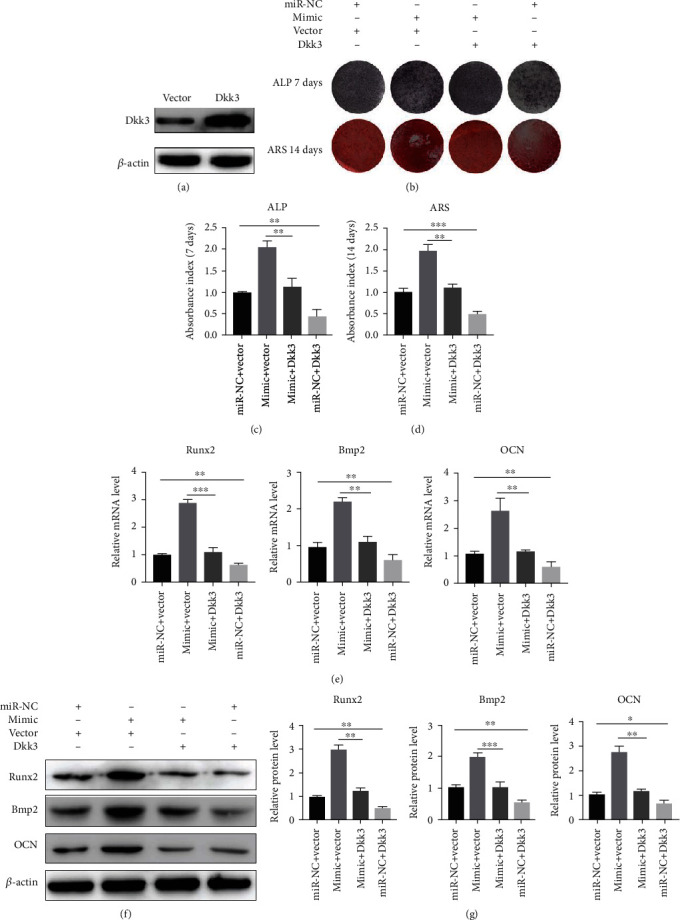
miR-129-5p regulated BMSC osteogenic differentiation via repressing Dkk3. (a) pcDNA-Dkk3 or pcDNA-vector was transfected into BMSCs. The expression of Dkk3 was analyzed by western blot. (b) ALP and ARS staining of BMSCs following miR-129-5p mimic transduction or overexpression of Dkk3 during osteogenic differentiation. (c, d) Absorbance index of ALP and ARS was calculated. mRNA (e) and protein (f) levels of osteoblast-related genes Runx2, Bmp2, and OCN were detected by quantitative RT-PCR and western blot, respectively. *β*-Actin was used as an internal control. (g) Semiquantitative analysis of protein expression levels of Runx2, Bmp2, and OCN. All data were expressed as means ± SD. ^∗^*p* < 0.05, ^∗∗^*p* < 0.01, and ^∗∗∗^*p* < 0.001.

To further confirm the role of Dkk3 as a mediator between miR-129-5p and osteoblast differentiation, osteogenesis with Dkk3 overexpression was performed. Transfection efficiency of pcDNA-Dkk3 vector was detected by western blot (Figure 7(a)). ALP and ARS data showed a significant increase in ALP activity and calcium nodule formation with miR-129-5p overexpression; however, Dkk3 overexpression could repress the effect of miR-129-5p on osteogenic differentiation (Figure 7(b)). To investigate the biological function of miR-129-5p and Dkk3 in osteogenesis, we detected the protein and mRNA levels of osteogenic markers in BMSCs transfected with miR-129-5p mimic or pcDNA-Dkk3. Consistently, RT-PCR and western blot results both showed that miR-129-5p mimic markedly increased Runx2, Bmp2, and OCN expression after osteogenic differentiation, while overexpression of Dkk3 repressed the promotion effect of miR-129-5p (Figures 7(e) and 7(f)). All these results indicated that miR-129-5p enhanced osteoblast differentiation via repressing Dkk3.

### 3.8. miR-129-5p Activated the Wnt/*β*-Catenin Pathway and Its Downstream Transcript Factors in BMSCs

**Figure 8:**
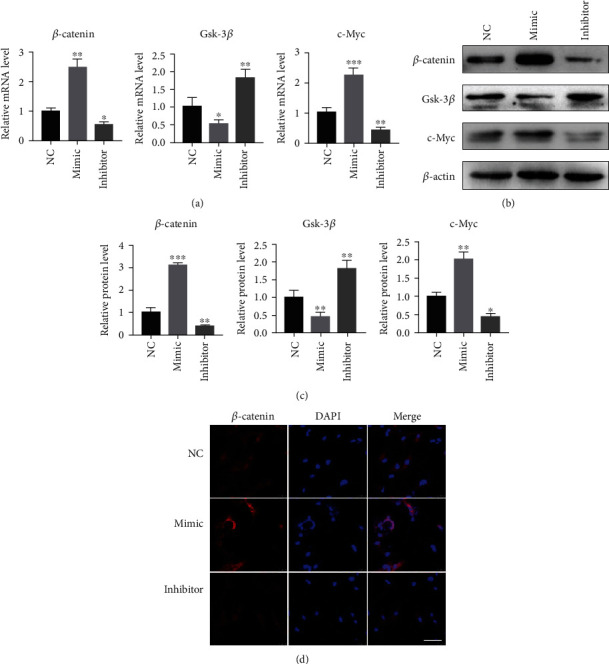
miR-129-5p activated the Wnt/*β*-catenin pathway and its downstream transcription factors in BMSCs. The mRNA (a) and protein (b) expression of *β*-catenin, Gsk-3*β*, and c-Myc were determined by quantitative RT-PCR and western blot in BMSCs transduced with a lentiviral vector containing miR-129-5p mimic, miR-129-5p inhibitor, or control (NC) lentivirus. (c) Quantitative analysis of protein expression levels of *β*-catenin, Gsk-3*β*, and c-Myc. (d) Immunofluorescence assays showing the expression and location of *β*-catenin in the indicated BMSCs. Scale bars, 50 *μ*m. All assays were repeated at least three times. Data were shown as means ± SD (*n* = 3).

We moved forward to investigate the mechanism of osteoblast differentiation promoted by miR-129-5p. The canonical Wnt/*β*-catenin pathway has been proven to be vital for bone homeostasis and BMSC osteogenic differentiation. To explore whether miR-129-5p influenced the process of osteogenesis through the Wnt/*β*-catenin pathway, relative key proteins in BMSCs treated with miR-129-5p mimic, miR-129-5p inhibitor, or control miRNA were investigated. Results showed that mRNA (Figure 8(a)) and protein expression (Figures 8(b) and 8(c)) levels of *β*-catenin, Runx2, and c-Myc were both significantly elevated by the overexpression of miR-129-5p and inhibited by the knockdown of miR-129-5p. Moreover, our data also demonstrated that the mRNA (Figure 8(a)) and protein (Figures 8(b) and 8(c)) expression of glycogen-synthetase-kinase-3*β* (Gsk-3*β*) were obviously lower in the mimic group compared with those in the NC group. Immunofluorescence staining demonstrated that the expression of *β*-catenin was also highest in the miR-129-5p mimic group (Figure 8(d)). We demonstrated by several methods that miR-129-5p activated the Wnt/*β*-catenin pathway and had a positive effect on the activities of its downstream molecules, which suggested that the regulation of miR-129-5p on osteoblast differentiation was probably through the Wnt/*β*-catenin pathway.

### 3.9. Dkk3 Interacted *β*-Catenin Directly

**Figure 9:**
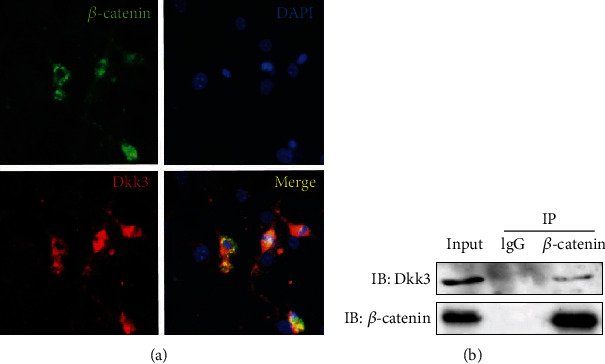
The interaction of Dkk3 and *β*-catenin. (a) Immunofluorescent localization showed the overlapping area of *β*-catenin and Dkk3 protein. Scale bars, 25 *μ*m. (b) Coimmunoprecipitation using anti-IgG (negative control) and anti-*β*-catenin showing interaction of Dkk3 with *β*-catenin in C3H10T1/2 cells. All assays were repeated at least three times. Data were shown as means ± SD (*n* = 3).

Previous reports have validated *β*-catenin, the key molecule of the Wnt signaling pathway, to be vital for bone homeostasis and BMSC osteogenic differentiation. Dkk3 may regulate osteogenic differentiation, but the molecular mechanism is still unclear. Based on above researches, we hypothesized that Dkk3 might connect *β*-catenin during osteogenesis. To further verify our hypothesis, we first applied immunofluorescent staining to trace the location of Dkk3 and *β*-catenin. Our results showed the overlapping of *β*-catenin and Dkk3 in C3H10T1/2 (Figure 9(a)). Then, we performed coimmunoprecipitation experiments and revealed that Dkk3 could bind to *β*-catenin in C3H10T1/2 cells (Figure 9(b)). Among these, we predicted certain relationship between Dkk3 and *β*-catenin.

## 4. Discussion

Bone defects have high incidence due to trauma, tumors, or congenital factors, which lead patients to suffer from muscle atrophy, bone mass reduction, and increased risk of fracture [23]. The vigorous development of regenerative medicine contributes to the repair of bone defects, but the regulatory factors in osteogenic differentiation and bone formation are still unclear. Our results demonstrated, for the first time, a novel role of the miR-129-5p/Dkk3 axis in regulating osteogenic differentiation of BMSCs and bone formation.

Osteogenic differentiation and bone regeneration are a long-term and complex biological process, which are regulated by genetic and epigenetic factors. Previous studies showed that, in addition to transcription factors, osteogenesis was tightly controlled by miRNAs, which may be an upstream mediator of transcriptional modulators. Arfat and colleagues described that mechanosensitive miR-208a-3p inhibited bone formation by targeting ACVR1, which is a regulator of the BMP pathway [24]. Maria reported that miRNAs (miR-21-5p, miR-129-5p, and miR-378-5p) were upregulated in progenitor cells in response to physical exercise [18]. miR-129-5p was reported to involve in gastric cancer, adipogenesis, and organ injury. A recent study demonstrated that miR-129-5p suppressed gastric cancer cell proliferation, migration, and invasion by selectively inhibiting COL1A1 [21]. miR-129-5p could significantly inhibit adipocyte differentiation through autophagy and may be a target for the treatment of obesity [25]. The study also revealed that miR-129-5p alleviated acute kidney injury via targeting the HMGB1/TLRs/NF-*κ*B pathway [26].

MicroRNAs play different roles in different tissues and cells during osteogenic differentiation [27]. miR-20a promoted osteogenic differentiation of human bone mesenchymal stem cells via Bmp or Wnt signaling [28, 29]. Contrary to the above research, Zhou et al. reported that miR-20a inhibited osteogenic differentiation of 3T3-L1 and C3H10T1/2 cells by targeting Tgfbr2 [30]. Researches about the function of miR-129-5p on osteogenesis still remain limited. Yin et al. found that miR-129-5p inhibited osteogenic differentiation of MC3T3-E1 cells and miR-129-5p inhibitor ameliorated menopause osteoporosis in C57BL6 mice [31]. However, another research demonstrated that human bone marrow mesenchymal stem cells were induced differentiation into osteoblast in vitro after overexpression of miR-129-5p [19]. These controversial reports may result from unstandardized protocols in different laboratories. In our study, we found that miR-129-5p expression was upregulated during osteogenic differentiation of both BMSCs and C3H10T1/2 cells. Moreover, our results revealed that miR-129-5p enhanced BMSC osteogenic differentiation in vitro and accelerated the reparative bone regeneration in a murine model of critical-size calvaria defect. Our data proved that miR-129-5p is a positive regulatory factor to osteoblast differentiation and bone formation.

The Wnt signaling pathway plays key roles in stimulating osteoblast generation and decreasing osteoclast differentiation [32], which is modulated by several different families of secreted regulators [33]. Our study suggested that miR-129-5p activated the expression of *β*-catenin and activities of downstream transcription factors of the Wnt/*β*-catenin pathway Runx2 and c-Myc. However, they are not direct target genes of miR-129-5p. To explore the mechanism of miR-129-5p in regard to osteoblast differentiation and bone formation, we searched for possible targets of miR-129-5p in molecules related to Wnt/*β*-catenin pathway regulation. Intriguingly, we found that miR-129-5p selectively targets Dkk3 through bioinformatics analyses and the subsequent use of a dual-luciferase reporter system.

**Figure 10:**
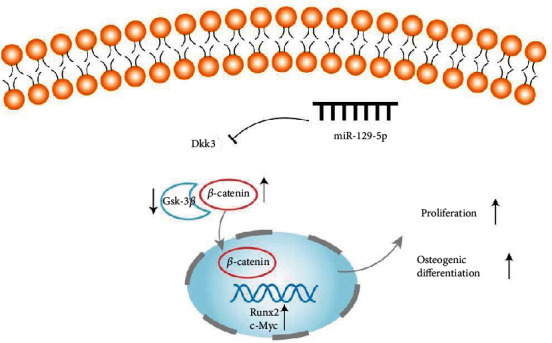
Schematic diagram of miR-129-5p/Dkk3 axis in osteogenic differentiation of BMSCs.

Dkk is a family of cysteine-rich proteins comprising at least four different forms (Dkk1, 2, 3, and 4), which are identified as exerting antagonist activity. Dkk1, 2, and 4 can bind Wnt coreceptor Lrp5/6 and Kremen proteins to antagonize Wnt signaling [34]. However, Dkk3 is a secreted protein and its role in the Wnt/*β*-catenin pathway is still elusive [35]. Yin et al. reported that Dkk3 was highly expressed in old mice, which lead to age-related muscle atrophy [36]. That indicated that Dkk3 may be related to aging-related diseases such as osteoporosis. Zhang et al. also found that Dkk3 negatively regulated the osteogenic differentiation of rat dental follicle cells [37]. Similarly, Aslan et al. reported the expression of Dkk3 protein might have inhibitory effects on osteogenic differentiation, but little effect on chondrogenesis [38]. Consistent with previous studies, our results demonstrated that overexpression of Dkk3 attenuated the osteogenic differentiation in BMSCs. Since *β*-catenin is a core component of the Wnt signaling and acts as a core function of this pathway, we further explored the relationship of Dkk3 and *β*-catenin. A correlation analysis demonstrated that Dkk3 expression levels were negatively correlated with *β*-catenin protein levels in BMSCs. Immunofluorescent staining and coimmunoprecipitation experiments revealed that Dkk3 directly interacted with *β*-catenin C3H10T1/2 cells. Without Wnt signaling activation, free *β*-catenin in cytoplasmic was low and then phosphorylated by a multiprotein destruction complex (APC, GSK3b, CK1, and PP2A), marking *β*-catenin for degradation [39]. Tang et al. reported that Smad7 promoted cell-cell adhesion by stabilizing *β*-catenin, protecting *β*-catenin from phosphorylation and degradation [40]. We supposed that Dkk3 may influence multiprotein destruction complex for free *β*-catenin degradation.  Figure 10 shows a schematic diagram of miR-129-5p/Dkk3 axis in osteogenic differentiation of BMSCs. However, the detailed molecular mechanisms of how Dkk3 regulates the Wnt/*β*-catenin pathway still require further study.

## 5. Conclusion

In summary, the present study indicated that miR-129-5p promoted osteogenic differentiation of BMSCs and then accelerated bone regeneration via targeting Dkk3. Accordingly, miR-129-5p/Dkk3 may represent a potential target for the future treatment for bone defect or bone loss. Further studies are needed to explore the exact action of Dkk3 on Wnt/*β*-catenin and how to apply miR-129-5p/Dkk3 to clinical treatment to repair bone defect.

## Figures and Tables

**Table 1:** RNA primer sequence.

Genes	Primers	Sequences (5′-3′)
Runx2	Forward	CCTTCAAGGTTGTAGCCCTC
	Reverse	GGAGTAGTTCTCATCATTCCCG
OCN	Forward	GGACCATCTTTCTGCTCACTCTGC
	Reverse	TCCTGCTTGGACATGAAGGCTTG
Bmp2	Forward	AGTAGTTTCCAGCACCGAATTA
	Reverse	CACTAACCTGGTGTCCAATAGT
*β*-Catenin	Forward	TGCCGTTCGCCTTCATTATGGAC
	Reverse	TGGGCAAAGGGCAAGGTTTCG
Gsk-3*β*	Forward	TGGTAGCATGAAAGTTAGCAGA
	Reverse	CTCTCGGTTCTTAAATCGCTTG
c-Myc	Forward	GGTTTGCCTCTTCTCCACAG
	Reverse	TCCTGTACCTCGTCCGATTC
*β*-Actin	Forward	CTACCTCATGAAGATCCTGACC
	Reverse	CACAGCTTCTCTTTGATGTCAC
miR-129-5p	Forward	CTTTTTGCGGTGTGGGCTTGC

## Data Availability

All data generated and/or analyzed in this study are included in this published article.

## Abbreviations

BMSCs: Bone marrow mesenchymal stem cells
ALP: Alkaline phosphatase
ARS: Alizarin red staining
MSCs: Mesenchymal stem cells
FBS: Fetal bovine serum
Micro-CT: Microcomputed tomography
BV/TV: Bone volume to tissue volume
BMD: Bone mineral density.
